# Polyphenols from Grape Pomace: Functionalization of Chitosan-Coated Hydroxyapatite for Modulated Swelling and Release of Polyphenols

**DOI:** 10.1021/acs.langmuir.1c01930

**Published:** 2021-12-14

**Authors:** Giacomo Riccucci, Sara Ferraris, Camilla Reggio, Antonella Bosso, Gissur Örlygsson, Chuen H. Ng, Silvia Spriano

**Affiliations:** † Politecnico di Torino, Corso Duca degli Abruzzi 24, 10129 Torino, Italy; ‡ Consiglio per la ricerca in agricoltura e l’analisi dell’economia agraria—Centro di Ricerca Viticoltura ed Enologia, via P. Micca 35, 14100 Asti, Italy; § Innovation Center Iceland, Árleynir 2-8, 112 Reykjavík, Iceland; ∥ Genis hf., Adalgata 34, 580 Siglufjördur, Iceland

## Abstract

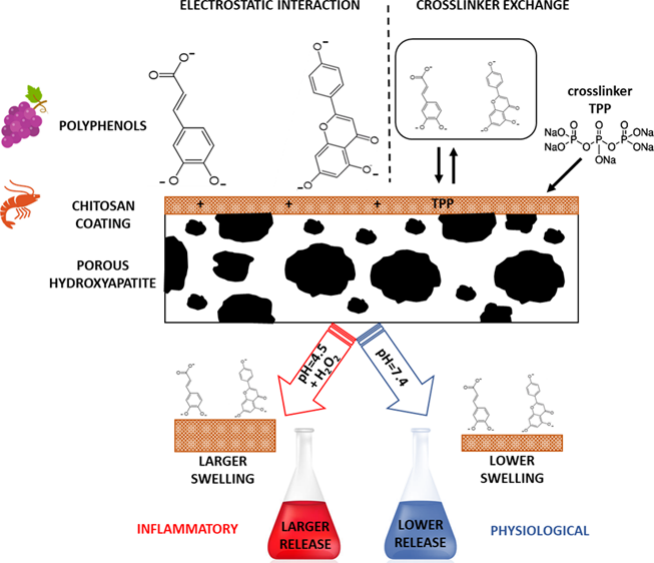

Chitosan is known for its specific antibacterial mechanism and biodegradability, while polyphenols are known for their antioxidant and anti-inflammatory properties: coupling these properties on a surface for bone contact, such as hydroxyapatite, is of great interest. The system developed here allows the combination of hydroxyapatite, chitosan, and polyphenol properties in the same multifunctional biomaterial in order to modulate the host response after implantation. Crosslinked chitosan is used in this research to create a stable coating on hydroxyapatite, and then it is functionalized for a smart release of the polyphenols. The release is higher in inflammatory conditions and lower in physiological conditions. The properties of the coated and functionalized samples are characterized on the as-prepared samples and after the samples are immersed (for 24 h) in solutions, which simulate the inflammatory and physiological conditions. Characterization is performed in order to confirm the presence of polyphenols grafted within the chitosan coating, the stability of grafting as a function of pH, the morphology of the coating and distribution of polyphenols on the surface, and the redox reactivity and radical scavenging activity of the functionalized coating. All the results are in line with previous results, which show a successful coating with chitosan and functionalization with polyphenols. Moreover, the polyphenols have a different release kinetics that is faster in a simulated inflammatory environment compared to that in the physiological environment. Even after the release tests, a fraction of polyphenols are still bound on the surface, maintaining the antioxidant and radical scavenging activity for a longer time. An electrostatic bond occurs between the negative-charged polar groups of polyphenols (carboxyls and/or phenols) and the positive amide groups of the chitosan coating, and the substitution of the crosslinker by the polyphenols occurs during the functionalization process.

## 1 Introduction

The challenging aim of this study is to obtain a bioactive material for bone contact applications (e.g., bone substitution or implants fully made or coated by hydroxyapatite), based on hydroxyapatite (HAp), with a coating of chitosan grafted with polyphenols: the coating is able to release the polyphenols in a smart delivery way, with a larger release in inflammatory conditions, useful for the control of the inflammatory body response after implantation. The system combines the HAp, chitosan, and polyphenol properties in the same multifunctional biomaterial. In this way, it is possible to modulate the host response to the implant by tailoring the bioactivity, antibacterial activity, and anti-inflammatory activity at the implant site.

HAp is a well-known biocompatible ceramic material; it is the main inorganic component of the hard tissue in the human body.^1^ HAp is osteoconductive and osteoinductive providing an interface where bone cells can easily adhere, grow, and differentiate.^2^ Furthermore, synthetic HAp induces the precipitation of natural HAp when in contact with physiological fluids (bioactivity). In medicine, HAp is currently used in bone contact applications as a bulk material,^3^ as a coating for metal implants,^4^ or in polymer-based composites.^5^

Chitosan is a polysaccharide obtained from chitin by deacetylation, and it is known to be highly biocompatible.^6^ The amino group is the functional group that differentiates chitosan from natural chitin, giving interesting chemical and biological properties.^7^ First of all, it exhibits antimicrobial activity based on the cationic nature of the amino groups,^6,8−10^ which avoid the risk of infection and biofilm formation. The biocompatibility tests underline a connection with the degree of deacetylation (DD): biocompatibility is higher at lower DD. Furthermore, chitosan has an impact on the inflammatory response like other polysaccharides; the immune response of chitosan depends on its DD, molecular weight, ionic charge, and solubility.^11^ Chitosan or its oligosaccharide degradation products have also recently been shown to have a bone remodeling effect in vivo, accelerating osteogenesis.^12^ For the high-interest properties listed here, chitosan is often used for different medical uses and in contact with human tissues.^11−15^

Polyphenols are a large group of natural molecules present in many plants (e.g., grapes) and are one of the most studied biomolecules.^16^ The family of polyphenols includes a great and diverse range of substances containing one or more phenol groups: a hydroxyl-substituted benzene ring. Polyphenols contain both elementary compounds and complex compounds, such as polymerized polyphenols, formed from elementary substances.^17^ All polyphenols are classified into two main categories: phenolic acids and flavonoids. The most interesting characteristic for biomedical applications is their antioxidant activity: flavonoids and phenolic acids are able to scavenge and inactivate free radicals and oxygen species.^18−20^ The properties of polyphenols can lead to a better integration of bone implants, optimizing the implants’ efficiency when there is a risk of chronic inflammation or infection and a decrease in the number of failures.^21^

The properties of polyphenols are widely exploited as pure molecules and mixtures in the cosmetic and nutraceutical fields, and the current research directions are investigating their application in the biomedical field in combination with biomaterials for wound healing,^22^ tissue regeneration,^23^ or bone contact applications.^24,25^ The combination of chitosan and polyphenols allows a synergic coupling of their properties as already outlined in the literature.^26^ Sun et al.^27^ evaluated chitosan functionalization with polyphenols, and the results showed that the antimicrobial activity of chitosan is improved after functionalization. In addition, the presence and release of polyphenols add large antioxidant capabilities to the chitosan coating.

The present work focuses on the combination of chitosan and polyphenols within a coating on HAp, which is still an unexplored way to couple these molecules on a surface. The study involves first crosslinking of the chitosan coating with tripolyphosphate^28^ to regulate the swelling of chitosan both at the inflammatory and physiological pH. Crosslinking is therefore relevant to modulate the following release of polyphenols. In this work, a natural extract of polyphenols from grape pomaces was used to get the advantages of a mixture of phenolic compounds (phenolic acids and flavonoids) and their synergic action.^29,30^ The term polyphenols includes, as previously discussed, a large family of molecules. In this work, for simplicity, the term will be used to describe the extract which is characterized by a mixture of molecules (mainly phenolic acids, flavonoids, and condensed tannins) obtained from red grapes as described in refs (24) and (25). The selected approach is also in line with the responsible use of resources and exploitation of local sources and waste from the food industry value chain (shrimp shells from the shellfish industry and pomaces from wine production).

In this paper, the chemical and physical properties of sintered HAp coated with crosslinked chitosan and then functionalized with polyphenols are reported to evaluate the presence, amount, distribution, bond type, and chemical reactivity of the grafted polyphenols. A release test in phosphate-buffered saline (PBS) and in a simulated inflammatory condition is performed to study the eventual release of the grafted polyphenols in different aqueous media (used as simplified biological conditions).

## 2 Materials and Methods

### 2.1 Sample Preparation

Hydroxyapatite (HAp; Ca_10_(PO_4_)_6_(OH)_2_) was prepared from α-tricalcium phosphate (TCP; α-Ca_3_(PO_4_)_2_) and tetracalcium phosphate (TTCP; Ca_4_(PO_4_)_2_O) purchased from Himed Ltd., USA. The powders were mixed thoroughly at a molar ratio of 2 (TCP)/1 (TTCP). An aliquot of excess ultra-pure water was added to the mixture, and the slurry was kept overnight at 40 °C to allow HAp formation. The excessive water was dried out to obtain cakes that were ground into powder. The powder (0.60 g) was weighed into a 13 mm diameter mold and pressed at 1400 N to obtain 2 mm-thick disks. The green state disks were placed on an aluminum oxide surface and sintered at 1200 °C for 10 h to reach the desired consolidation. The final diameter of the disks was 12 mm. After the preparation of the samples, their surface was cleaned: the protocol included an immersion in acetone for 5 min and then three washes of 5 min in deionized water in an ultrasonic bath. The samples were dried before chitosan coating. The descriptions and acronyms of the samples are reported in Table 1.

**Table 1:** Table of Acronyms

name and description	acronym
hydroxyapatite	HAp
freeze-dried extract of polyphenols	sample P
pomace flour	PM
functionalization solution	TRIS/HCl + CaCl_2_
hydroxyapatite coated with chitosan and crosslinked	HAp_CH
hydroxyapatite coated with chitosan, crosslinked, and functionalized with polyphenols	HAp_CH_P
hydroxyapatite coated with chitosan, crosslinked, functionalized with polyphenols, and used for the release test at pH 7.4	HAp_CH_P_PBS
hydroxyapatite coated with chitosan, crosslinked, functionalized with polyphenols, and used for the release test at pH 4.5	HAp_CH_P_H_2_O_2_

### 2.2 Polyphenol Extract from Barbera Grapes

The fermented pomace of organic Barbera grapes was sampled at racking off after soft pressing. The whole pomace (skins + seeds) was dried in a ventilated oven (48 h, 35 °C) and then milled (coffee grinder, 1 min) to obtain a pomace flour (PM, powder). The extraction of polyphenols was performed according to a previous work:^32^ the PM was extracted with ethanol/water (1:1), and the extraction ratio was 1:6 w/v flour/solvent. The extract was centrifuged at 18 °C for 20 min at 2880*g* (Centrifuge 5810 R Eppendorf—Hamburg, Germany), and the supernatant was separated from the solid residue and freeze-dried (sample P). The descriptions and acronyms of the samples are reported in Table 1.

### 2.3 Chitosan and Tripolyphosphate Solutions

The chitosan powder is supplied by Genis hf. (Iceland) as reported in ref (31). The chitosan powder was dissolved in a solution of 1% acetic acid to obtain a concentration of 0.75% w/v. The chitosan solution was stored in a refrigerator at 4 °C, and it was brought to room temperature 1 h before coating deposition.

Sodium tripolyphosphate (TPP) (technical grade, 85%, Sigma-Aldrich, Germany) was added to water to obtain a concentration of 15% w/v. The pH of the solution was adjusted at 6 with diluted phosphoric acid (*ortho*-phosphoric acid 85%, extra pure, Merck, Germany) to control protonation of the amino groups of chitosan, promoting a better crosslinking with TPP.^33,34^

### 2.4 Chitosan Coating

The solutions described in Section 2.3 were used for the coating protocol of HAp. The HAp samples (prepared as described in Section 2.1) were immersed in the chitosan solution for 5 min at room temperature and then left to dry at room temperature overnight to form the chitosan coating. The coating was crosslinked by immersing the coated samples in the TPP solution for 1 h at room temperature: crosslinking of the chitosan coating is performed to avoid excessive swelling. Three washes with ultra-pure water were performed: one for 1 min and twice for 90 min and dried at room temperature. The crosslinking protocol was adapted starting from Shu and Silva’s work.^34^ The descriptions and acronyms of the samples are reported in Table 1.

### 2.5 Functionalization of the Coated Samples with Polyphenols

An aqueous solution was prepared by dissolving CaCl_2_ (0.292 g), HCl 1 M (39 mL), and tris(hydroxylmethyl)aminomethane (TRIS) (6.118 g) in 1 L of ultra-pure water. The TRIS and HCl buffer pH was at 7.4.^25,35,36^ The freeze-dried polyphenols were added to the aqueous solution reaching a concentration of 5 mg/mL with magnetic stirring for 1 h at room temperature. Polyphenols are photosensitive and could be damaged or degraded by exposition to light; thus, the whole procedure was carried out under dark condition. The samples of HAp coated with chitosan and crosslinked (as described in 2.4) were immersed in 5 mL of the polyphenol solution for 3 h at 37 °C in an incubator. Each sample was washed twice in ultra-pure water and dried at room temperature. The descriptions and acronyms of the samples are reported in Table 1.

### 2.6 Release Protocols

Two solutions were used to perform the release tests, one to simulate a physiological environment and the other one to simulate an inflammatory environment. For the preparation of PBS solution, which simulates a physiological environment, a PBS tablet (PBS, tablet, Sigma-Aldrich) was dissolved in 200 mL of ultra-pure water to yield 0.01 M phosphate buffer with pH 7.4. A protocol reported in the literature was followed to simulate the inflammatory environment (labeled H_2_O_2_):^37,38^ hydrogen peroxide (30% w/v, PanReac Applichem) was added to PBS to reach a concentration of 0.05 M, which simulates an inflammation process,^39,40^ and then HCl solution was employed to obtain the acidic environment at pH 4.5.

The samples coated with chitosan, crosslinked, and functionalized with the polyphenols (HAp_CH_P—as described in 2.5) were soaked in 15 mL of physiological solution or simulated inflammatory solution, at 37 °C, under the dark condition for 24 h. At the end, the samples were washed and stored under the dark condition until the characterization tests.

### 2.7 FTIR–ATR Analysis

The measurements were performed with a Fourier transform infrared spectroscopy–attenuated total reflectance (FTIR–ATR) instrument (Nicolet iS50 FTIR Spectrometer, Thermo Scientific, USA) to study the functional groups on the surface of the samples. The wavenumber was set in the range between 4000 and 600 cm^–1^, carrying out 32 scans for each measure with a resolution of 4 cm^–1^.

### 2.8 X-ray Photoelectron Spectroscopy

The chemical analysis was performed on HAp_CH and HAp_CH_P through X-ray photoelectron spectroscopy (XPS, PHI 5000 VersaProbe, Physical Electronics, USA). Initially, a portion of surface of 400 × 400 μm was chosen to perform a survey, considering a range of 0–1200 eV. Then, the high-resolution measurement was carried out in the C, O, N, and Ca regions. The spectra were referenced to the C 1s peak at 284.80.

### 2.9 Morphological Analysis with Scanning Electron Microscopy Images

The samples for scanning electron microscopy (SEM) analysis were covered by a thin gold layer (∼10 nm). After the deposition of the conductive layer, the measurements were performed with a Carl Zeiss instrument (Leo Supra 25 FESEM, Zeiss, Germany). Two different magnifications were used: 100× and 2500×, with the voltage set at 5 kV and the aperture at 30 μm.

### 2.10 Total Polyphenols by Folin–Ciocalteu Test

In the case of liquid samples, the Folin–Ciocalteu reagent (0.5 mL) and sodium carbonate (1.5 mL) were added to the test solution, formed by the solution to be analyzed (2 mL) and ultra-pure water (6 mL), to reach alkaline pH. The product of the reaction was quantified by measuring the absorbance at 760 nm through a UV–vis spectrometer (UV2600, Shimadzu, Japan); the total amount of polyphenols is expressed as gallic acid equivalent (GAE).^41−43^ In the case of modified protocol for solid samples, the specimens were immersed in 8 mL of ultra-pure water, and the F&C reagent and sodium carbonate were added in the same quantity used in the previous case. The test samples were left under the dark condition for 2 h at room temperature before measuring the absorbance. The concentration was calculated by using a calibration curve with the equation
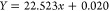
where *Y* corresponds to the measured absorbance and *x* corresponds to the total polyphenol concentration. The calibration curve was determined using the measured absorbance of six gallic acid solutions with known concentrations (0.001, 0.0025, 0.005, 0.01, 0.02, 0.03, and 0.04 mg/mL).

Then, GAE is expressed as mg/mm^2^ when it was measured on a solid sample, to raise the readability of the results; it is expressed in mg in the case of release solutions.

### 2.11 Radical Scavenging Activity by DPPH Test

The measurement of radical scavenging activity (RSA) was based on the protocol explained in previous studies.^44,45^ The free-radical DPPH (2,2-diphenyl-1-picrylhydrazyl, Sigma-Aldrich) was dissolved in ethanol with a concentration of 0.4 w/v, sonicated in an ultrasonic bath, filtered with a 0.25 μm filter, and diluted in ethanol (1:10). Then, each sample was immersed in 3 mL of DPPH solution for 24 h under the dark condition at room temperature. The measurement of absorbance at 515 nm was performed at 4 and 24 h. For quantifying the RSA, the following equation was used:
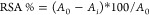
where *A*_0_ is the reference value of absorbance at 515 nm of DPPH solution and *A*_*i*_ is the value of absorbance of the solution with the sample analyzed with UV at 515 nm.

### 2.12 Fluorescence Microscopy

Polyphenols reflect in the red range of the light,^46,47^ showing autofluorescence properties. Measurements of fluorescence were carried out with a confocal microscope (LSM 900, Zeiss, Germany) equipped with a filter in the red range. Each image was taken at 200× and with an exposure time of 1 s.

### 2.13 Zeta Potential Titration Measurements

The measurement of zeta potential titration was performed with an electrokinetic analyzer (SurPASS, Anton Paar, Austria). An electrolyte solution of KCl 0.001 M was used to determine the zeta potential values as a function of pH. The pH was adjusted through the addition of 0.05 M HCl for the acidic titration or 0.05 M NaOH for the alkaline titration. The samples were placed with parallel surfaces at a distance of 100 μm, and the same couple of samples was subjected first to acidic titration and then to basic titration; in both cases, the starting point was at pH 5.5. The tested samples are the substrate (HAp), the coated and crosslinked samples (HAp_CH), and the coated, crosslinked, and functionalized samples (HAp_CH_P).

### 2.14 UV–Vis Spectroscopy

To evaluate the presence of grafted polyphenols on the solid samples, UV–vis analyses were performed by a UV–vis spectrophotometer (UV2600, Shimadzu, Japan) equipped with an integration sphere for recording the diffuse reflectance spectra. The diffuse reflectance baseline was recorded with BaSO_4_ powder. The spectrum was collected in the wavelength range of 200–700 nm.

### 2.15 Statistical Analysis of the Data

The results of F&C and DPPH tests were statistically compared by analysis of variance (ANOVA) and Tukey’s test with JMP16 (JMP, SAS Institute Inc., Milan, Italy). The significant level of Tukey’s test was set at *p* < 0.05.

## 3 Results and Discussion

### 3.1 FTIR–ATR

**Figure 1:**
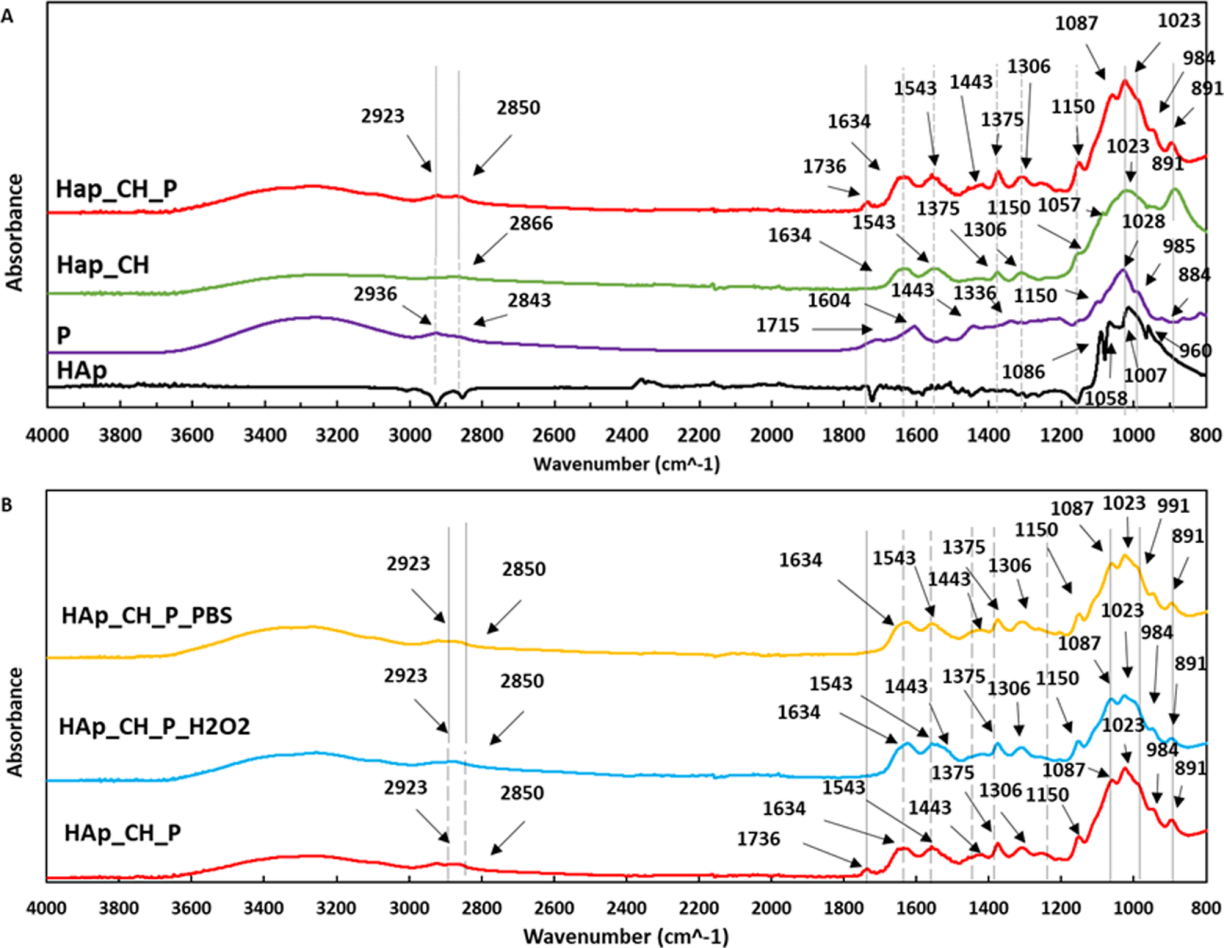
FTIR–ATR spectra of (A) uncoated hydroxyapatite (HAp), freeze-dried polyphenols (P), and coated hydroxyapatite (HAp_CH) and (B) functionalized samples immersed in PBS for 24 h (HAp_CH_P_PBS) and functionalized samples immersed in a simulated inflammatory solution for 24 h (HAp_CH_P_H_2_O_2_). The spectrum of the coated samples after functionalization (HAp_CH_P) is reported both in (A,B). Dotted lines are used to represent the characteristic peaks of chitosan and solid lines for the polyphenol’s peaks, and lines with half and half indicate that chitosan and polyphenols present the same peak/band.

Throughout the analysis of the FTIR spectra, the presence of the crosslinked chitosan coating on HAp (HAp_CH) and of the polyphenols, after functionalization (HAp_CH_P), was verified on the different surfaces. The spectra of the extract of the polyphenols (sample P) and of the substrate (HAp) were used as a reference. The curves are presented in Figure 1A, and the peaks are interpreted thanks to previous works.^48−57^

The spectrum of the grape freeze-dried extract (Figure 1A—sample P) shows the characteristic peaks of the polyphenols’ functional groups. The curve has a large band between 3600 and 3000 cm^–1^, which is representative of OH stretching. The peaks at 2936 and 2843 cm^–1^ are connected, respectively, to the asymmetrical and symmetrical vibrations of CH_2_. The bending vibration of C=O in the carboxyl group shows a peak at 1715 cm^–1^; there is also a peak connected to the stretching of the carbonyl group at 1604 cm^–1^ and it is characteristic of flavanols. The peaks at 1514 and 1443 cm^–1^ are due to the C–C stretching of aromatic rings. Plane bending of CH_3_ caused the peak at 1336 cm^–1^. The three peaks at 1150, 1103, and 1028 cm^–1^ are connected to the stretching of the C–O bonds in the alcohol groups. Below 1000 cm^–1^, there are peaks of glycosylated polyphenols.

HAp (Figure 1A—sample HAp) shows the characteristic peaks of the v3 normal modes of vibration of the phosphate ion in HAp around 1000 cm^–1^. The representative band of the vibration of the phosphate is evident at 960 cm^–1^.

The spectrum of chitosan-coated HAp (Figure 1A—sample HAp_CH) shows the main peaks typical of chitosan. The characteristic bands of the OH group between 3500 and 3100 cm^–1^ are evident; then, in this range, there is also an overlapped peak of the NH stretching vibration. The peak at 2866 cm^–1^ is caused by the CH_2_ vibration in aliphatic compounds. Plane bending of the secondary amides is connected to the peaks at 1634 and 1543 cm^–1^. There are peaks at 1375 cm^–1^ and 1306, respectively, related to both the CH_3_ deformation and vibration of the C–N bond in the amines and C=O group. The three peaks at 1150, 1057, and 1023 cm^–1^ are connected to the stretching of the C–O bonds in the alcohol groups. Below 1000 cm^–1^, there is the peak at 881 cm^–1^ connected to the stretching of the P–O–P bridge vibration.

The spectrum of the coated samples after functionalization (Figure 1A—sample HAp_CH_P) reveals the presence of polyphenols on the chitosan coating with an increase of intensity of the OH broad band (3600–3000 cm^–1^) and the peak of bending of C=O that is slightly shifted (1736 cm^–1^); this peak is connected to the C=O stretching in esters (polyphenols). The peak at 1634 cm^–1^ is connected to the C=O stretch of the chitosan amides. Instead, the characteristic peak of the carboxylic group of polyphenols at 1604 cm^–1^ shows a decrease of intensity after the interaction with chitosan. To evaluate the decrease of the peak at 1604 cm^–1^, the ratio between the intensity of the peaks of the carboxylic and OH groups (3450 cm^–1^) was calculated, both for samples P and HAp_CH_P. The ratio is 0.56 for sample P, and it decreases below 0.07 after functionalization. It can be supposed that these groups are involved in the chemical electrostatic bond between polyphenols and chitosan within the coating.^58,59^ The intensity of the TPP peak is clearly decreased after functionalization with the polyphenols (891 cm^–1^).

In Figure 1B, the spectra of the samples HAp_CH_P analyzed after soaking in PBS (HAp_CH_P_PBS) and in a simulated inflammatory solution (HAp_CH_P_H_2_O_2_) are reported. The broad band of OH (3600–3000 cm^–1^) is almost unchanged after soaking, while the peak of bending of C=O (1736 cm^–1^) is no more observable according to a partial release of polyphenols during soaking in both scenarios.

On the basis of the obtained data, FTIR is a technique suitable for confirming the effective coating of the HAp substrate with chitosan and the presence of the crosslinker TPP in the as-prepared coating (HAp_CH). Furthermore, FTIR provides evidence of the effective functionalization of the chitosan coating with polyphenols (HAp_CH_P), and it suggests the role of the carboxylic group of the polyphenols in the chemical bond with chitosan, as well as of the replacement of TPP after functionalization. A partial release of polyphenols after soaking is also suggested. All these results are confirmed through the following characterization.

### 3.2 XPS Analysis

Table 2 reports the atomic composition of the samples after coating with chitosan (HAp_CH) and after functionalization of the coating with the polyphenols (HAp_CH_P); the chemical composition of the freeze-dried extract of polyphenols (sample P) is reported as the reference.

**Table 2:** Surface Chemical Composition Using XPS Survey Analysis

	atomic (%)						
sample	carbon	oxygen	phosphorus	nitrogen	calcium	sodium	silicon
HAp_CH	42.6	42.1	5.2	5.1	2.7	1.7	0.6
HAp_CH_P	69.0	27.4		3.0	0.1		0.5
P	69.0	28.7		1.5	0.2		

The chemical composition of the chitosan-coated HAp surface is mainly constituted by carbon, oxygen, phosphorus, and nitrogen, as expected.^60,61^ The high percentages of carbon, oxygen, and nitrogen are connected to the chemical composition of chitosan, whereas phosphorus is connected to the presence of TPP, which is used as a crosslinker by creating ionic bonds between the NH^3+^ ions of chitosan and the P_3_O^5–^ ions of TPP. The presence of calcium is due to the substrate of HAp; sodium and silicon can be considered as contaminants (Table 2). In the freeze-dried polyphenols, high percentages of carbon and oxygen are detected, as expected.^25^ Low amount of nitrogen and calcium can be considered as contaminants in the case of polyphenols.

After the functionalization of the coating with the polyphenols, the carbon percentage significantly increases, confirming the presence of the phenolic compounds. The detected percentages of oxygen, nitrogen, and calcium significantly decrease, as well as phosphorus and sodium disappear. All the observed variations of the atomic percentages underline the presence of polyphenols, a lower amount of TPP on the surface, and the absence of the substrate exposed on the outermost surface of the samples (HAp_CH_P).

**Figure 2:**
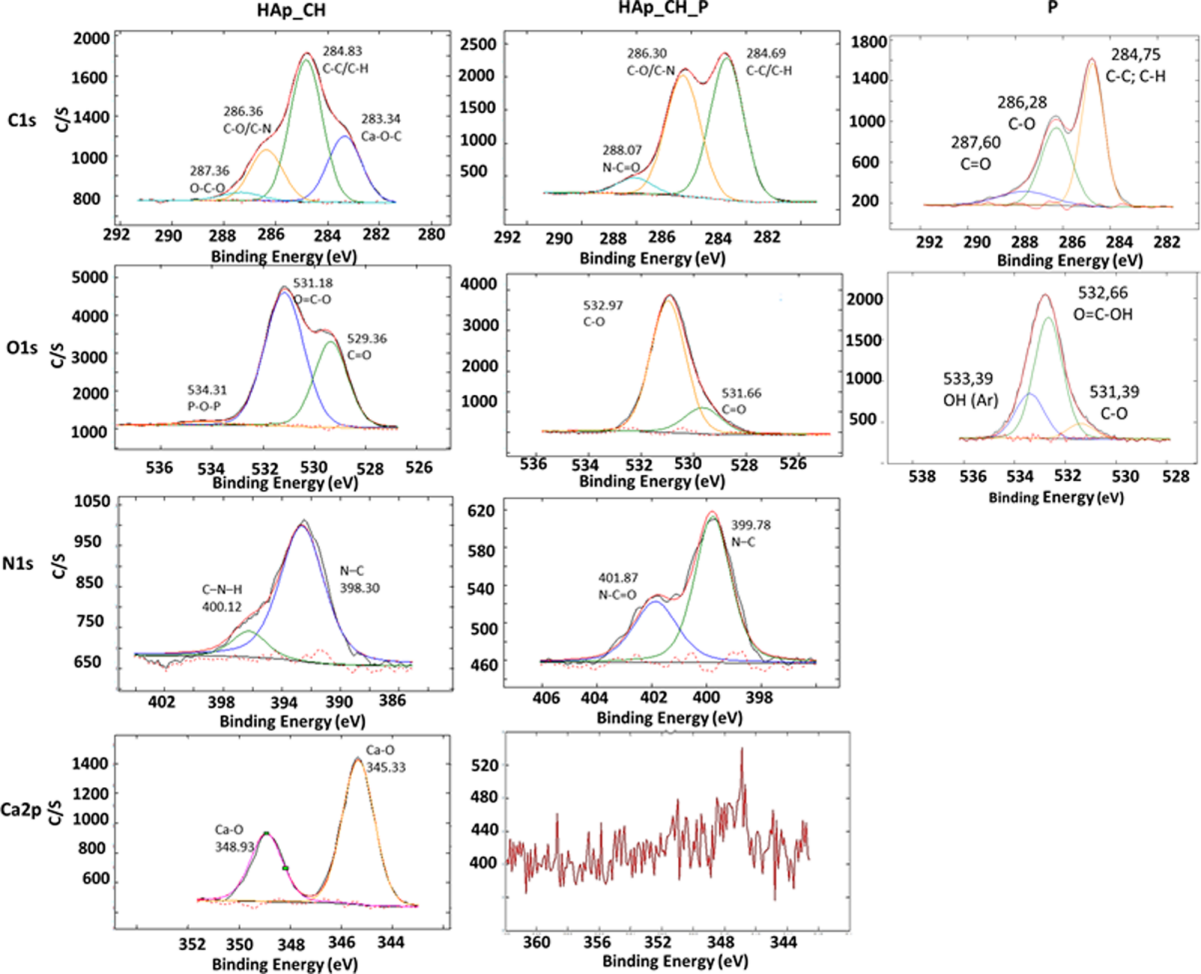
XPS high-resolution spectra of HAp_CH, HAp_CH_P, and sample P. High resolutions in C, O, N, and Ca regions.

Before the functionalization with the polyphenols (HAp_CH), the high-resolution spectrum of the chitosan coating presents four peaks in the carbon region at 283.34, 284.83, 286.36, and 287.36 eV (Figure 2). The second peak is connected to C–C or C–H,^62^ and the third and fourth peaks are assigned to C–O or C–N and O–C–O.^63^ Finally, the first peak at 283.34 eV is connected to the bonds between carbon and metals or metal ions with an oxygen bridge.^24^ In the oxygen region, the observed peaks are at 529.36, 531.18, and 534.31 eV. The first two peaks indicate the C=O and O=C–O groups, and the last peak shows the presence of TPP because it is connected to the P–O–P bonds.^64^ The study of the nitrogen region reveals two peaks at 398.30 and 400.12 eV connected, respectively, to C–N of the amine terminal group^65,66^ and C–N–H.^63^ The calcium region has two peaks at 345.33 and 348.93 eV connected to the Ca–O bonds.^67^

The high-resolution spectrum of the freeze-dried polyphenols (sample P) reveals peaks at 284.75, 286.28, and 287.60 eV, which can be associated with C–C or C–H, C–O, and C=O, respectively. In the oxygen region, three peaks are visible at 531.39, 532.66, and 533.39 eV, which can be attributed to C–O, O=C–OH, and phenolic OH.^24^

After the functionalization of the coating with the polyphenols (HAp_CH_P), there is a clear variation of all the examined regions. In line with the survey analysis, in the region of calcium, there are no peaks, and in the oxygen region, the characteristic peak of P–O–P is not detected (Figure 2). In the carbon region, there are three peaks at 284.69, 286.30, and 288.07 eV assigned to C–C or C–H, C–O or C–N, and N–C=O, respectively.^68^ The peak at 286.30 eV increases in intensity, showing a major amount of C–O/C–N. Two peaks are found in the oxygen region connected to C–O and O=C–OH, the first is at 531.66 eV and the second is at 532.97 eV, showing a shift of the polyphenols peaks of 0.3 eV toward higher binding energies. The characteristic peaks of the aromatic OH group in the polyphenols (533.39 eV) is not detected after the functionalization. In the nitrogen region, there are two peaks at 399.78 and 401.87 eV connected to C–N and N–C=O.^68^ The spectrum indicates that calcium is not involved in the chemical bond of the polyphenols with the chitosan coating as it was when direct functionalization of HAp was performed without any coating.^25^ In the case described here, the link occurs between the negative-charged polar groups of polyphenols (carboxylic and/or phenols) and the positive amide groups of the chitosan coating; substitution of TPP as a crosslinker by the polyphenols can also be supposed, as discussed later on.

It can be concluded that the XPS data confirm the results obtained through FTIR analysis and the hypotheses above mentioned about the effectiveness of the coating and functionalization procedures.

### 3.3 Morphological Analysis with SEM

The study of the morphology of the HAp surface before and after coating with chitosan was done with SEM on the HAp and HAp_CH samples.

**Figure 3:**
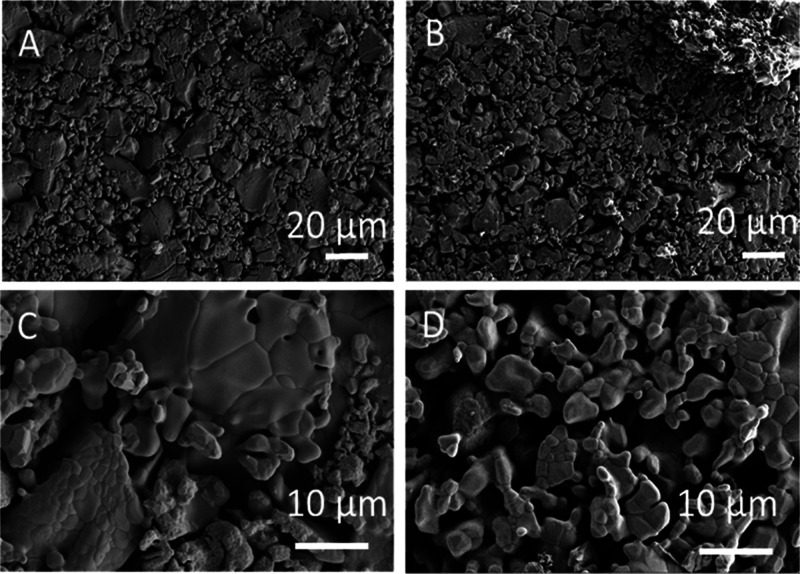
SEM analysis of uncoated (HAp) and coated hydroxyapatite (Hap_CH). (A) 500×: uncoated hydroxyapatite; (B) 500×: chitosan-coated hydroxyapatite; (C) 2500×: uncoated hydroxyapatite; and (D) 2500×: chitosan-coated hydroxyapatite.

The SEM images (Figure 3A–C) show a great porosity of the HAp samples and a large size distribution of the grains on the surface (Figure 3C). The comparison between before (Figure 3A) and after (Figure 3B) the chitosan coating indicates that the HAp surface morphology is maintained; thus, the chitosan coating adheres to the substrate following the topography of the surface. The great porosity increases the surface area, leading to a great quantity of chitosan exposed on the surface and suitable for the functionalization.

### 3.4 Folin–Ciocalteu Test

**Figure 4:**
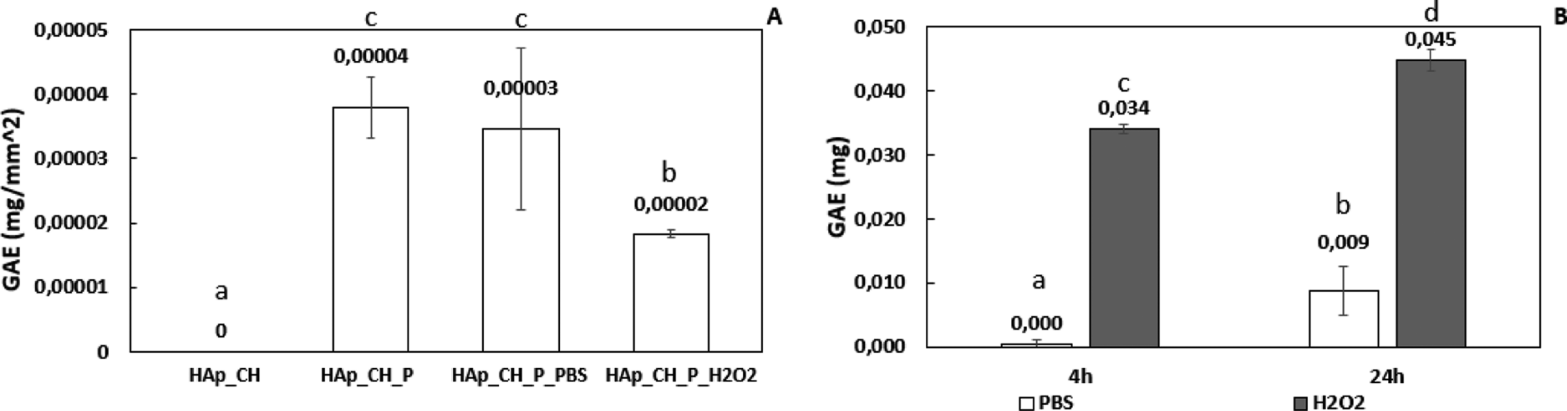
(A) GAE index of solid samples (before and after surface functionalization and after 24 h of soaking in the release solutions) and (B) GAE index (expressed as mg of GAE) of the release solutions (15 mL) of PBS or simulated inflammatory environment (H_2_O_2_) after 4 and 24 h of soaking; the bars that share the same letter are not significantly different (*p* < 0.05 calculated with Tukey’s test).

The F&C test was used to evaluate the quantity of the polyphenols in the chitosan coating, after the functionalization, and in the liquids of the release tests.^35^ A modified F&C test^35^ was used on the solid samples, and the results are reported in Figure 4A, while the traditional F&C test was applied to the release solutions, and the data are reported in Figure 4B. The F&C test is reported in the literature as the most suitable method to determine the grafting ratio of phenolic compounds to chitosan.^26^

As it is expected, the as-prepared coating of chitosan (HAp-CH) does not react with the F&C reagent, showing a null result of the test and no redox chemical activity. On the contrary, the formation of a colored compound, after the F&C test, leads to detectable absorbance at 760 nm (Figure 4A) in the case of the samples functionalized with polyphenols (HAp_CH_P). The value of GAE is 0.00004 mg/mm^2^ after functionalization, showing an effective functionalization. The one-way ANOVA test confirms that there is a statistically significant difference of the GAE value of the coating before and after the functionalization. After 24 h of immersion in the release solutions, the GAE value shows no statistically significant reduction, if the release environment is at pH 7.4 (*p* > 0.05), but there is a clear and statistically significant decrease of GAE after immersion in an inflammatory environment at pH 4.5 (Figure 4A).

A supplementary study of the release solutions (for 4 and 24 h) with the traditional F&C test (Figure 4B) confirms that a higher release occurred from the functionalized samples soaked in a solution at acidic pH with hydrogen peroxide (HAp_CH_P_H_2_O_2_) with respect to soaking in PBS (HAp_CH_P_PBS). As last, there is no complete release of polyphenols in the first 4 h in the analyzed solutions: the release is increasing over time (at 24 h of soaking) in both cases. In the literature, many authors suggested that the pH is lower and the release of polyphenols is higher. If the polyphenols are electrostatically bound with chitosan, the release of polyphenols is due to erosion and diffusion processes.^69^ Silva-Weiss et al. have found that at pH 4.5, there is an electrostatic interaction between gallic acid and chitosan because the COOH group is exposed and can be linked to the NH_3_^+^ group of chitosan.^70^ Other authors also found that crosslinking with TPP can modulate the release of polyphenols and that the chemical electrostatic bond is due to the NH_3_^+^ group of chitosan and the OH group of polyphenols.^71^

These data give a second confirmation of the effectiveness of the functionalization procedure and evidence a difference in the kinetics of the release of the polyphenols in different chemical environments with a “smart” and larger release of polyphenols by the chitosan coating in a solution mimicking the inflammatory conditions. This was one of the purposes of this work considering the relevance of inflammatory condition in the healing process around an implanted biomaterial.

### 3.5 DPPH Test

The DPPH test allows us to determine the radical scavenging power of a sample surface in contact with a free radical. The test was performed at 4 and 24 h to evaluate the scavenging capability as a function of time. The values are expressed as the percentage of DPPH inhibition (RSA %), and they are related to a DPPH solution without any samples as a control.

**Figure 5:**
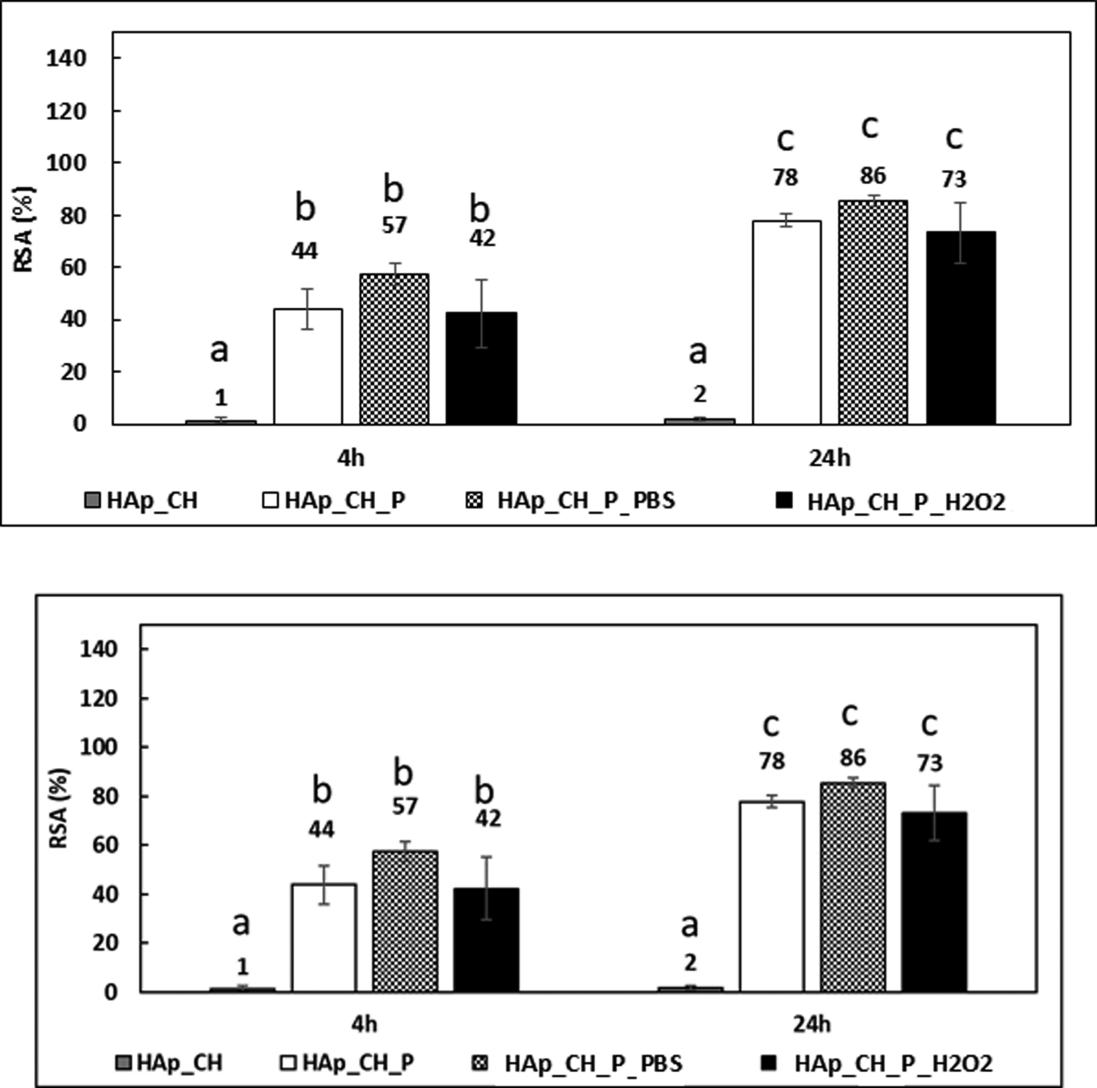
RSA obtained with the DPPH test, the values are expressed in percentage, and they were evaluated at 4 and 24 h of soaking in the DPPH solution. The bars that share at least one letter are not significantly different (*p* < 0.05 calculated with Tukey’s test).

The samples coated with chitosan and crosslinked with TPP (HAp_CH) do not show any radical scavenging property; in fact, the RSA is negligible both at 4 and 24 h (Figure 5) with no statistically significant difference (*p* > 0.05), as expected. After the functionalization with the polyphenols (HAp_CH_P), the samples show a much higher RSA (*p* < 0.05), already clear at 4 h when the samples stabilize a large number of free radicals in the solution (RSA = 44%). At 24 h, there is a further significant (*p* < 0.05) increase of the RSA percentage, showing an increase of 34%. The RSA of the surfaces is still clearly measured after 24 h, and it indicates that the soaking during the DPPH test does not nullify the scavenging capability of the grafted polyphenols. The values of the DPPH test, performed at 4 and 24 h, of the samples tested after the release for 24 h in different environments (HAp_CH_P_PBS and HAp-CH-P_H_2_O_2_) show results not statistically different (*p* > 0.05) from the as-prepared functionalized samples, confirming the presence of an outermost grafted layer of polyphenols on the soaked samples. It must be underlined that this test is probably sensitive to the outermost layer of the surface and not to the total amount of grafted polyphenols within the coating, as in the F&C test.

The F&C and DPPH tests provide evidence of the redox and radical scavenging ability of the functionalized chitosan. This effect is one of the main aims of this research.^72^ It could be of great relevance on a bone implant surface where an excessive inflammatory response can compromise osseointegration and induce implant loosening.^73^ It is expected that the low percentage of DD of the investigated chitosan induces a higher biocompatibility, promoting proliferation of bone cells.^74^ Moreover, the selected chitosan should act as an anti-inflammatory and antimicrobial agent because of the molecular weight.^75^ The antibacterial action of chitosan could be synergic with the properties of the grafted polyphenols: a specific biological investigation of this functionalized coating will be performed to investigate this point.

### 3.6 Zeta Potential Titration Measurements

**Figure 6:**
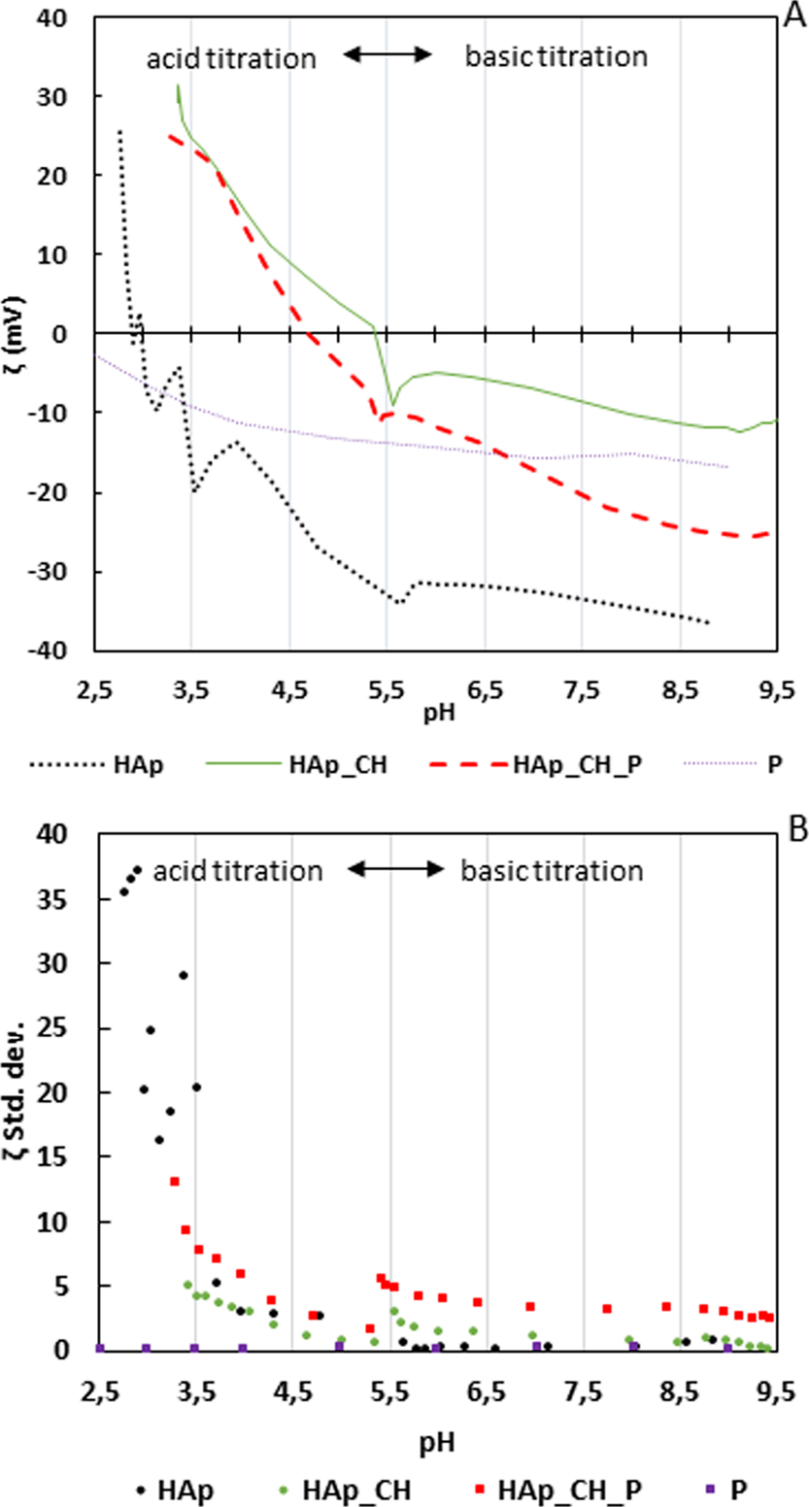
(A) Zeta potential titration curves of uncoated hydroxyapatite (HAp), coated hydroxyapatite (HAp_CH), functionalized with polyphenols samples (HAp_CH_P), and polyphenols in the functionalization solution (P). (B) Standard deviation of zeta potential titration curves reported in (A).

The zeta potential titration curves of the substrate (HAp), the coated (HAp_CH), and the functionalized samples (HAp_CH_P) are reported in Figure 6A. The zeta potential titration of a surface as a function of pH allows us to investigate the isoelectric point (IEP), exposition of functional groups on it, and its chemical stability as a function of pH. The titration curve of the polyphenols (sample P) is reported as a reference (Figure 6A).

The curve of HAp shows a negative zeta potential of the surface in almost all the explored range with an IEP as low as 2.75. This is due to the presence of the acid OH groups on HAp: they are partially deprotonated at any pH higher than 2.75 and completely deprotonated at pH higher than 5.5 when the curve reaches a plateau. The high standard deviation below pH 3.5 is due to the high reactivity of HAp below this pH (Figure 6B), which releases ions into the solutions decreasing the stability of the measured zeta potential.

Polyphenols in solution (with the same ionic strength of the functionalizing solution) have a negative zeta potential for the whole measured range. The value of zeta potential decreases with two variations of the slope of the curve: the first one is around pH = 4 (due to deprotonation of the phenolic functional groups) and the second one is around pH = 7 (due to the carboxylic groups). At pH 7, the curve reaches a plateau because the OH groups of polyphenols are completely deprotonated. At pH 7.4, when the functionalization occurs, polyphenols have a negative zeta potential value, and both the phenolic and carboxylic groups are deprotonated.

The curve of the chitosan-coated samples shows the IEP around pH 5.5 (Figure 6); this value is the balance between the presence of the deprotonated acid groups and protonated amino groups exposed by chitosan.^76,77^ The shift of the IEP toward the basic range with respect to the curve of HAp indicates that there is a chitosan layer which covers the HAp surface and a prevalence of the protonated NH_3_^+^ groups of chitosan below pH 5.5. On the other hand, when the HAp_CH curve becomes negative, it indicates that the deprotonated carboxylic groups are prevalent with respect to the amino groups. When the curve reaches a constant negative plateau (at alkaline pH higher than 8), it indicates that the carboxylic groups are completely deprotonated. Despite a slight increase of standard deviation below pH 4 (Figure 6B), the curve has low standard deviation values, showing a great stability of the coated surface in the full range of the measure. It indicates that the chitosan layer is stable and shows small swelling (mainly at pH lower than 4) because of crosslinking with TPP. Moreover, it acts as a protection against the chemical reactivity of HAp at low pH.

After functionalization with polyphenols, the IEP shifts toward lower pH, about at pH 4.7 (Figure 6), because of the grafted polyphenols and the acid hydroxyl groups exposed. The deprotonation of the OH groups of polyphenols with the increase of pH leads to a decrease of the value of the zeta potential with respect to the unfunctionalized sample. In the acid titration, the acidic curve shows a significant increase of standard deviation in the range of pH 5–7 (Figure 6B). Below pH 4, again the standard deviation increases. These results further confirm the presence of polyphenols on the functionalized surface and suggest the release of polyphenols from the surface at the physiological and, even more, inflammatory pH (4.5–5.5). This is because of the trend of standard deviation in this range of pH: a higher standard deviation of the zeta potential is related to a change of surface charge, as it occurs during releasing of the grafted polyphenols. The chemical bonds between chitosan and polyphenols, as it is deduced from XPS (Section 3.2) and FTIR-ATR (Section 3.1) results, are due to an electrostatic attraction between the carboxylic or phenolic groups of polyphenols and the amino group of chitosan. The larger release of polyphenols at low pH provides evidence of an important role of the carboxylic groups and aromatic hydroxyl groups of the polyphenols and electrostatic interactions in the grafting process: a larger release occurs when these groups are protonated. This also suggests that the phenolic acids are selectively grafted to chitosan among the different compounds in the natural extract (phenolic acids, flavonoids, and condensed tannins). A further hypothesis is that TPP acts as a leaving group: during functionalization, it is partially substituted by the polyphenols. This reaction could explain the absence of calcium and phosphorous in the XPS analysis and the increase of standard deviation at pH lower than 4 in the zeta potential titration curve of the functionalized surface: the coating has a lower crosslinking degree after functionalization and larger swelling occurs at low pH. This explanation is in agreement with the literature: it is reported that tannic acid interacts with the chitosan chains, acting as a crosslinking agent.^78^ The crosslinking ability of tannic acid is related to electrostatic interactions, ester linkages, and hydrogen bonds.^70^ The natural extract of polyphenols used in this work contains condensed tannins and can act in a similar way.

### 3.7 Fluorescence Microscopy

The unfunctionalized and functionalized samples were observed by fluorescence microscopy and compared to study the distribution of the polyphenols in the chitosan coating. Previous studies demonstrated the autofluorescence of polyphenols in the red field^43,47,79^ and utility of the fluorescence microscopy for observing the presence and distribution on the surface of the autofluorescent grafted molecules.

**Figure 7:**
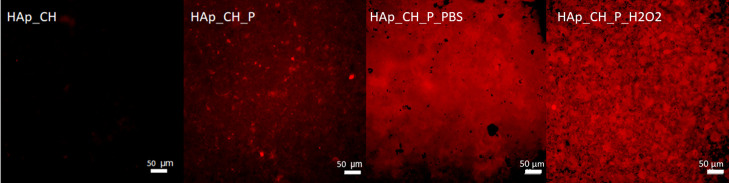
Fluorescence intensity in the red range of coated HAp (HAp_CH), functionalized samples (HAp_CH_P), functionalized samples after the release test in PBS (HAp_CH_P_PBS), and functionalized samples after the release test in a simulated inflammatory solution (HAp_CH_P_H_2_O_2_).

Figure 7 shows the different intensity of fluorescence of the unfunctionalized and functionalized samples. The image of HAp_CH has an almost null autofluorescence, indicating that chitosan has no autofluorescence capability in the red field. The red signal of HAp_CH_P shows the presence of polyphenols in the chitosan coating. The polyphenols are distributed in a homogenous way on the whole surface; then there are some aggregates of polyphenols which are distinguishable as red spots. The spots have a large size range, from few microns until tens of microns.

After immersion in the release solutions (HAp_CH_P_PBS and HAp_CH_P_H_2_O_2_—Figure 7), the autofluorescent polyphenols are still well observable. As first, it indicates that the polyphenol release was not complete during soaking in both conditions: a quote of polyphenols is firmly bonded to chitosan and not easily released. This is in accordance with the DPPH test which does not reveal a significant change of the antioxidant properties of the outermost layer of the surface, too. These results underline the presence of active polyphenols on the tested surfaces, which conserve interesting properties after soaking in a simulated physiological or inflammatory environment. The difference with respect to the F&C results can be explained considering that this test is much more sensitive to the total amount of polyphenols in the whole coating,^48^ while DPPH and fluorescence microscopy are sensitive mainly to the outermost surface.

It can also be noted that after 24 h of soaking in PBS, the samples (HAp_CH_P_PBS) have a homogeneous layer of polyphenols without the evidence of any agglomerate (as it was before the soaking on HAp_CH_P). Probably, the polyphenols that are released during soaking at pH 7.4 in PBS are connected to the agglomerates which were visible by fluorescence microscopy on HAp_CH_P. The simulated inflammatory solution (HAp_CH_P_H_2_O_2_) causes a larger swelling of chitosan, which leads to a change of morphology of the surface: it has a much more porous appearance. This observation put in evidence that the swelling of chitosan, which is pH sensitive, has also a role in the “smart” release of polyphenols at a specific pH.

### 3.8 UV–Vis Spectroscopy

**Figure 8:**
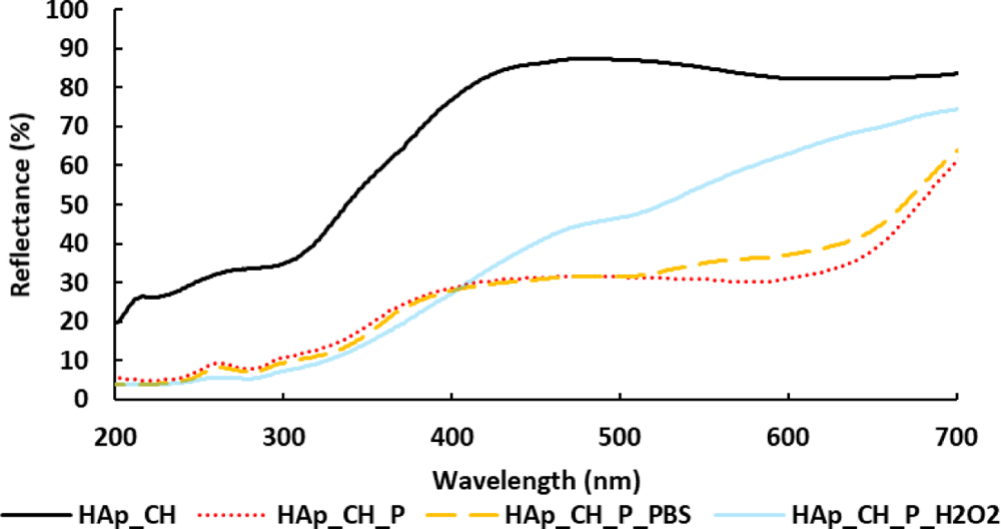
UV–vis spectra expressed in the reflectance mode of coated hydroxyapatite (HAp_CH), functionalized with polyphenol samples (HAp_CH_P), functionalized samples immersed in PBS (HAp_CH_P_PBS), and functionalized samples immersed in simulated inflammatory solution (HAp_CH_P_H_2_O_2_).

The visible spectrum of HAp_CH shows values of reflectance around 80% from 700 to 400 nm. In the UV range, the values decrease below 30% (Figure 8). The grafted polyphenols influence the trend of the previous spectrum. A decrease of reflectance is clearly visible on HAp_CH_P in all the explored wavelength range: it is connected to the shielding properties of the polyphenols of the visible and UV ranges. This reduction in the diffusive UV reflectance after functionalization, thanks to the shield capability of polyphenols, once again confirms that the here developed process of functionalization, as well as the here selected materials and polyphenol extract, allows effective polyphenol grafting. As last, it must be underlined that the grafting of polyphenols occurred in this work without any linker. This is not trivial considering the literature,^80^ where it is reported that some toxic reagents (such as EDC or EDAC) or instable radicals are often added to allow this type of grafting.^81^

The spectrum of the samples after immersion in PBS (HAp_CH_P_PBS) is similar to that of the as-prepared HAp_CH_P, confirming, through the shield effect, the presence of polyphenols, which is also in line with the previous results (fluorescence in Figure 7, F&C in Figure 5, and DPPH in Figure 4). It can be finally concluded that soaking in a PBS solution at pH 7.4 for 24 h does not significantly alter the polyphenol layer. On the other hand, the grafted polyphenols are subjected to a significant release after soaking in a simulated inflammatory solution at acidic pH and in the presence of hydrogen peroxide: the change of the UV spectrum of HAp_CH_P_H_2_O_2_ is in line with the previous results (fluorescence images, F&C, and DPPH). The low reflectance in the UV range, caused by the grafted polyphenols, confirms again their presence after the release test in a simulated inflammatory environment, but the higher reflectance in the visible range provides evidence that they were partially released. It cannot be set, at this stage, if any selective release of some specific phenolic compounds occurs, but it will be investigated in the future.

## 5 Conclusions

The protocol used in this paper allows the functionalization of a crosslinked chitosan with polyphenols, forming a functionalized coating on HAp with improved properties. After functionalization, the coating demonstrates a slight decrease of chemical stability but relevant antioxidant and radical scavenging properties. In addition, it is demonstrated that the functionalized chitosan coating can modulate the release of polyphenols and enables a larger release in inflammatory conditions. In conclusion, the interaction with polyphenols and chitosan can be a possible solution to control the release of polyphenols in an inflammatory environment while maintaining an active antioxidant and radical scavenging properties.
